# Transradial versus transfemoral access for cardiac catheterization: a nationwide pilot study of training preferences and expertise in The United States

**DOI:** 10.1186/s12872-021-02068-5

**Published:** 2021-05-21

**Authors:** Khalid Changal, Mubbasher Ameer Syed, Ealla Atari, Salik Nazir, Sameer Saleem, Sajjad Gul, F. N. U. Salman, Asad Inayat, Ehab Eltahawy

**Affiliations:** 1 Department of Cardiovascular Medicine, University of Toledo, Toledo, OH USA; 2 College of Medicine and Life Sciences, University of Toledo, Toledo, OH USA; 3 Department of Cardiovascular Medicine, University of Kentucky, Bowling Green, USA; 4 Internal Medicine, St. Francis Medical Center, University of Illinois at Peoria, Peoria, USA; 5 Internal Medicine, Mercy St. Vincent Medical Center, Toledo, OH USA; 6 Department of Medicine, Khyber Teaching Hospital, Peshawar, Pakistan; 7 Professor and Program Director of Cardiovascular Medicine and Interventional Cardiology, University of Toledo, 3000 Arlington Ave., MS 1118, Toledo, 43614 OH USA

**Keywords:** Radial access, Femoral access, Campeau radial paradox, Radial first

## Abstract

**Background:**

The objective was to assess current training preferences, expertise, and comfort with transfemoral access (TFA) and transradial access (TRA) amongst cardiovascular training fellows and teaching faculty in theUnited States. As TRA continues to dominate the field of interventional cardiology, there is a concern that trainees may become less proficient with the femoral approach.

**Methods:**

A detailed questionnaire was sent out to academic General Cardiovascular and Interventional Cardiology training programs in the United States. Responses were sought from fellows-in-training and faculty regarding preferences and practice of TFA and TRA. Answers were analyzed for significant differences between trainees and trainers.

**Results:**

A total of 125 respondents (75 fellows-in-training and 50 faculty) completed and returned the survey. The average grade of comfort for TFA, on a scale of 0 to 10 (10 being most comfortable), was reported to be 6 by fellows-in-training and 10 by teaching faculty (p<0.001). TRA was the first preference in 95% of the fellows-in-training compared to 69% of teaching faculty (p 0.001). While 62% of fellows believed that they would receive the same level of training as their trainers by the time they graduate, only 35% of their trainers believed so (p 0.004).

**Conclusion:**

The shift from TFA to radial first has resulted in significant concern among cardiovascular fellows-in training and the faculty regarding training in TFA. Cardiovascular training programs must be cognizant of this issue and should devise methods to assure optimal training of fellows in gaining TFA and managing femoral access-related complications.

## Background

Coronary angiography and percutaneous coronary intervention [PCI] are cornerstones in the management of coronary artery disease. Traditionally, PCI was performed primarily via transfemoral access [TFA] due to large arterial size allowing for easier cannulation of the artery, manipulation of catheters, simultaneous placement of mechanical support devices, and shorter door-to-balloon times in the setting of ST elevation myocardial infarctions. Successful transradial angiography was first reported in 1989 by Canadian cardiologist Lucien Campeau [1, 2]. Shortly thereafter, Kiemeneij et al. compared femoral, radial, and brachial access sites in the ACCESS study and found that transradial access [TRA] was associated with the lowest percentage of complications compared to the femoral or brachial routes (0%, 2%, 2.3% respectively) [3, 4]. Over the last 30years, multiple observational and randomized studies comparing TFA and TRA have reported lower rates of bleeding and vascular complications with TRA [1, 5,14]. The decreased access site bleeding was shown to lower mortality in multiple subsets of patients undergoing PCI via TRA [1, 9,11, 15,26]. Furthermore, TRA was shown to reduce time to ambulation, improve patient comfort, and decrease overall costs and length of hospital stay [10, 16, 27,38].

Based on these and multiple other advantages shown with TRA, it has become the default access site for PCI in Europe, Asia, and the United States [16, 17, 19, 20, 39,41]. In the last decade, there has been an unprecedented initiative to move away from TFA and favor the Radial first approach. The adoption of TRA as the primary mode of access has raised concerns regarding proficiency with TFA, potentially jeopardizing outcomes when TFA is needed [19, 42,46]. This phenomenon has been termed the Campeau Radial Paradox [42]. Regardless of whether a paradoxical decline in outcomes with TFA truly exists, inadequate experience with transfemoral access and management of related complications remain legitimate concerns for trainers and trainees.

The purpose of this study was to assess the current training preferences, expertise, and practice with TFA and TRA amongst cardiovascular training fellows and teaching faculty in the academic cardiovascular, interventional, and advanced interventional training programs in United States.

## Methods

**Fig. 1:**
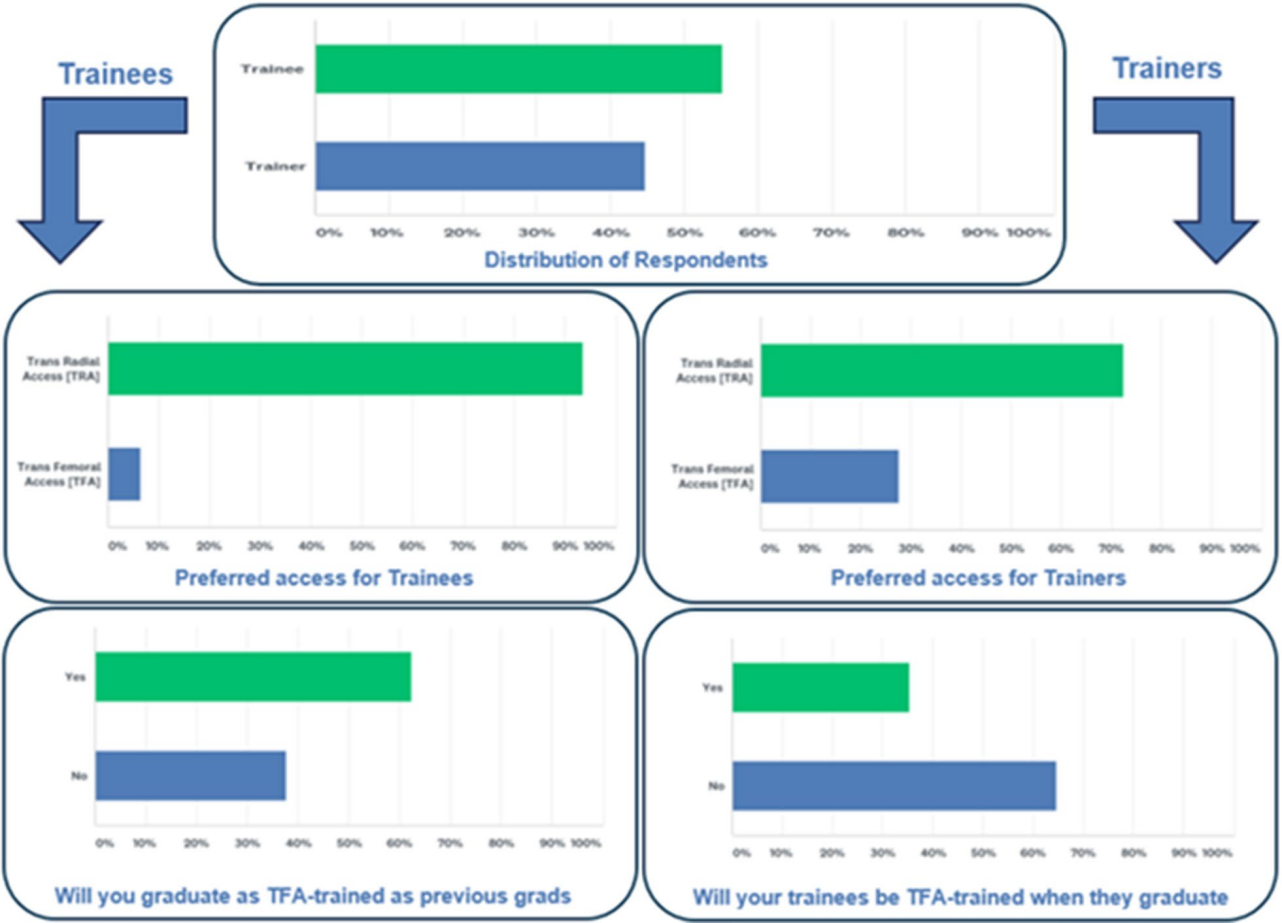
Distribution of respondents, their access preferences and perception of current training in transfemoral access

An online questionnaire to compare preferences, apprehensions, and practice for the two access options was designed. The questions addressed the clinical/academic settings, geographic location of practice, number of procedures performed, and number of questions about the volume and practice of TRA and TFA. Two independent reviewers audited this questionnaire to ensure there were no leading questions and all relevant aspects pertinent to access related decision making were covered. The questionnaire was emailed to 239 academic General Cardiovascular and 160 Interventional Cardiology training programs in the United States that were listed on FREIDA [Fellowship and Residency Electronic Interactive Database] (Fig.1).

The initial email was followed by two reminder emails 30days apart, allowing a total time of 90days to maximize responses. The answers were analyzed for significant differences between training fellows and teaching faculty using SPSS Version 20. Group differences were compared using the Pearson 2 or Fishers exact test for categorical variables, or the Student t test or the MannWhitney *U* test for continuous variables.*P*-values of 0.05 or less were considered statistically significant.

Institution review board of the University of Toledo Health Sciences determined that Ethics approval was not needed as no patient data was collected.

## Results

**Fig. 2:**
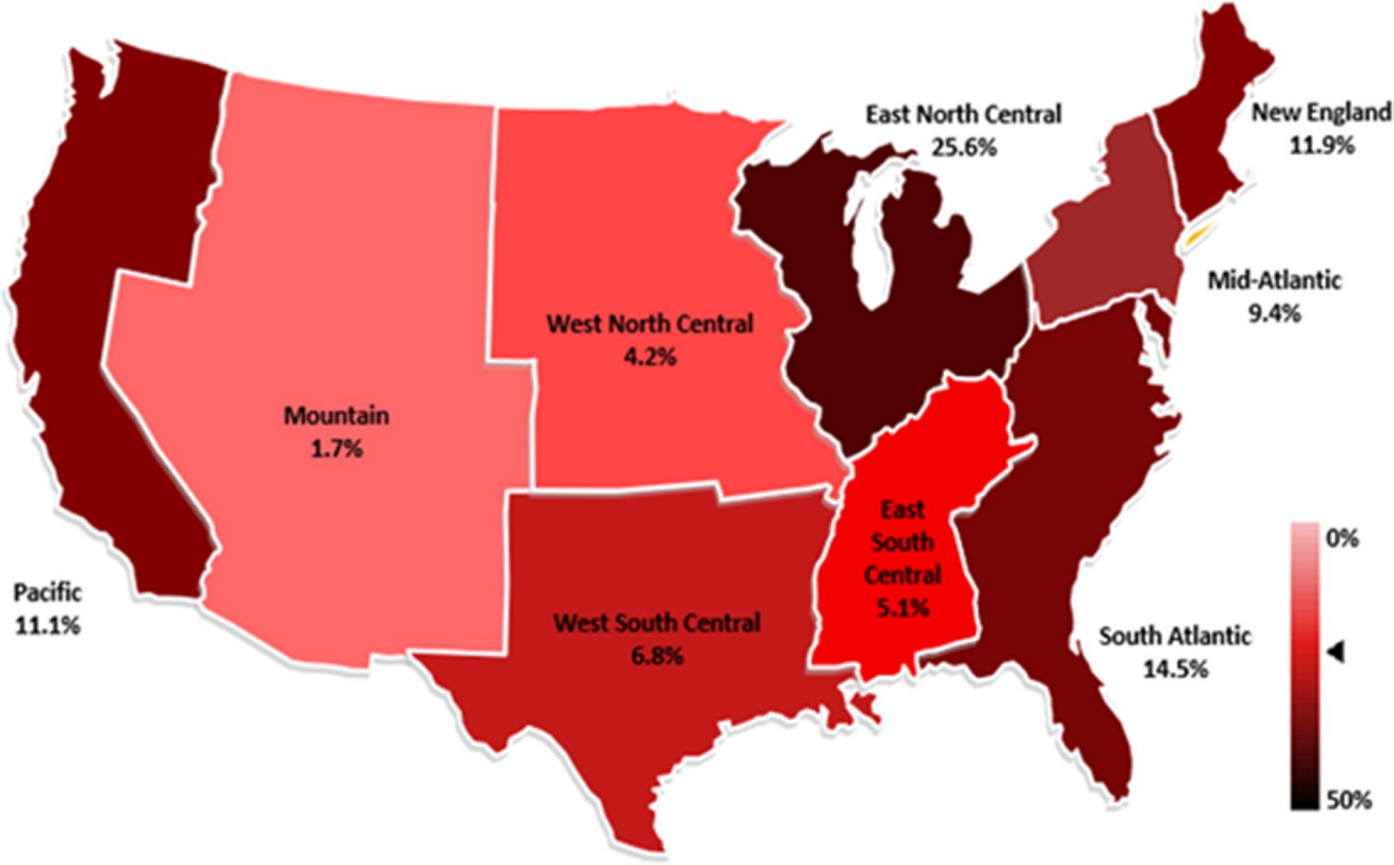
Mapped and color-coded geographical distribution of survey respondents (The figure is generated using an online software from surveymonkey.com and is freely available to use.)

125 cardiovascular physicians (75 fellows and 50 faculty) completed and returned the survey. The response rate as a proportion of email addresses invited to participate in the survey was 34%. Table 1 summarizes the sample of respondents in terms of their training site, geographical location (Fig.2) and their procedural exposure. The respondents were evenly distributed from all geographical locations in the USA.

**Table 1:** Training site, geographical location and procedural exposure of the respondents

Variable	Total % (n)	Trainee % (n)	Trainer % (n)	P value
Number of Respondents	100 (125)	60 (75)	40 (50)	
*Training Site Category*				
University Hospital	72 (90)	70.6 (53)	74 (37)	0.68
Community hospital	12 (15)	10.6 (8)	14 (7)	0.57
University affiliated	16 (20)	18.6 (14)	12 (6)	0.32
*Region in USA*				
New England	11.9 (14)	13.4 (9)	10 (5)	0.57
Middle Atlantic	9.4 (11)	16.4 (11)	0 (0)	0.002
East north central	25.6 (30)	28.3 (19)	22 (11)	0.44
West North central	4.2 (5)	4.4 (3)	4 (2)	1.0
South Atlantic	14.5 (17)	13.4 (9)	16 (8)	0.69
East South Central	5.1 (6)	5.9 (4)	4 (2)	1.0
West south central	6.8 (8)	8.9 (6)	4 (2)	0.46
Mountain	1.7 (2)	1.5 (1)	2 (1)	1.0
Pacific	11.1 (13)	7.4 (5)	16 (8)	0.15
*Procedures Performed*				
Coronary Angiography/PCI	98.2 (114)	98.5 (66)	98 (48)	0.82
Peripheral interventions	47.4 (55)	43.2 (29)	53 (26)	0.29
Coronary CTO	47.3 (54)	44.7 (30)	51.0 (24	0.51
Structural interventions	29.3 (34)	23.9 (16)	36.7 (18)	0.13

The average grade of comfort for TFA, on a scale of 0 to 10 (10 being most comfortable), was reported to be 6 by trainees and 10 by teaching faculty (p<0.001). The average proportion of daily transfemoral access was reported to be 34% by trainees and 35% by teaching faculty, the remainder being transradial (p 0.58). Operators who had managed more than 20 femoral complications in the previous one year were 3% among trainees versus 8% among trainers (p 0.26). While 24% of teaching faculty were comfortable enough with TFA that ultrasound guidance for it was not utilized, only 2% of fellows-in-training felt the same (p<0.001). TRA was the first preference in 95% of the fellows-in-training compared to 69% of teaching faculty (p 0.001). These findings are summarized in Table 2. Various possible reasons were investigated for chosen preference and respondents could choose more than one determinant for their respective choices. The results from that query are summarized in Table 2.

**Table 2:** Transfemoral Vs. Transradial access practices in Fellows-in-Training and teaching faculty

Variable	Total % (n)	Trainee % (n)	Trainer % (n)	P value
*Use of ultrasound for gaining TRA*				
US TRA every case	14 (15)	18 (11)	8 (4)	0.16
US only if initial assessment hints difficulty	43 (47)	44 (27)	41 (20)	0.72
US if pulse guided access fails once or twice	34 (37)	31 (19)	37 (18)	0.54
Never	10 (11)	6.5 (4)	14 (7)	0.18
*Use of ultrasound for gaining TFA*				
US TFA every case	50 (55)	56 (34)	43 (21)	0.18
If initial assessment hints difficult access	22 (24)	26 (16)	16 (8)	0.2
If pulse guided access fails once or twice	16 (18)	16 (10)	16 (8)	0.9
Never	12 (13)	2 (1)	24 (12)	<0.001
*Percentage of TFAs on an average day in the catheterization lab*				
Average TFA per day (Mean percent)	34.4	34	35	0.58
*Grade of expertise and comfort with access [Scale of 110; 10 being expert]*				
Self-graded expertise and comfort for TRA	8	7	9	<0.001
Self-graded expertise and comfort for TFA	7.8	6	10	<0.001
*Witnessed and managed TFA-related complications in preceding 12months*				
Less than 5	38 (42)	33 (20)	45 (22)	0.19
510	43 (47)	46 (28)	39 (19)	0.45
1115	11 (12)	13 (8)	8 (4)	0.41
1620	3 (3)	5 (3)	0 (0)	0.25
More than 20	5 (6)	3 (2)	8 (4)	0.26
*Preferred Access*				
TFA first	16 (18)	5 (3)	31 (15)	0.001
TRA first	84 (92)	95 (58)	69 (34)	0.001
*Reason for preference of access*				
Associated risk factor profile of access	72 (79)	75 (46)	67 (33)	0.35
Seen/done/taught more	49 (54)	62 (38)	33 (16)	0.002
Ability to maneuver	20 (22)	9 (5)	35 (17)	0.001
Preservation of radial conduits	3 (4)	0 (0)	8 (4)	0.04
Ability to upsize to larger bore	13 (14)	3 (2)	24 (12)	0.001
Ease of closure	64 (70)	69 (42)	57 (28)	0.20
Patient satisfaction	75 (83)	85 (52)	63 (31)	0.008
Training center radial first policy	40 (44)	51 (31)	26 (13)	0.01
High exposure to radial arterial lines	12 (13)	20 (12)	2 (1)	0.006
Low exposure to femoral arterial lines	12 (13)	21 (13)	0 (0)	0.001
*Future of TFA*				
Will remain just as frequent & viable	17 (19)	18 (11)	16 (8)	0.81
Will be entirely replaced	3 (3)	3 (2)	2 (1)	1.0
Frequency will reduce but will remain viable for certain cases	80 (88)	79 (48)	82 (40)	0.70
*Do you believe you [they] will get the same level/expertise of TFA training as your predecessors by the time you [they] graduate?*				
Yes	50 (55)	62 (38)	35 (17)	0.004
*Are you concerned about the quality/quantity of TFA training?*				
Yes very	23 (25)	11 (7)	37 (18)	0.002
Yes somewhat	34 (37)	36 (22)	31 (15)	0.54
Not concerned, satisfied	41 (45)	51 (31)	28 (14)	0.02
No, exposure is declining but TFA will soon be irrelevant	1.8 (2)	1.6 (1)	2 (1)	1.0
*Validity of the proposed Campeau Radial Paradox*				
Believe the paradox is Valid	63 (69)	61 (37)	65 (32)	0.62

When asked about the expected level of expertise in the future, 62% of fellows believed that they would receive the same level of training as their trainers by the time they graduate, only 35% of their trainers shared their optimism (p 0.004). A lower proportion of the trainees [11%] were overly concerned about the lack of TFA training compared to 37% of their trainers (p 0.002). 51% of the fellows were fully satisfied with the TFA training they were currently receiving compared to only 28% of the training faculty.

## Discussion

With a global trend towards TRA, the proficiency and comfort of operators and trainees with TFA has come into question. Although TRA is now the preferred method of access for diagnostic and therapeutic procedures, there are still specific patient populations and clinical situations that require TFA [19, 22, 47]. However, with unusually high rates of access site complications being observed in patients undergoing femoral PCI by default radial operators [32, 32, 32], many are questioning if evidence of the loss of transfemoral competency has begun to show [42, 48, 49]. These apprehensions are subjectively shown by our survey in which trainees reported a lower level of comfort [6/10] with TFA, compared to TRA [9/10] and 95% of the trainees chose TRA as their default access. The commonest reasons cited by trainees for radial preference were, in order;patient satisfaction, low complication profile, ease of closure and the training centers radial first policy.

In the midst of a dramatic shift from TFA to TRA, operator experience undoubtedly becomes a major determinant of outcomes [50, 51]. Based on our results, while daily use of TFA was quite comparable between trainees and trainers [34% vs. 35%], trainees reported a much lower exposure to the management of TFA related complications. We also queried the preference for ultrasound guidance for both radial and femoral access amongst respondents. Ultrasound guided TFA has been shown to reduce access site complications, and more than 98% of trainees reported they would use ultrasound for TFA. With less than 50% of trainers using ultrasound for femoral access and 24% "never" using ultrasound, there is a concern about the quality of teaching trainees are experiencing. This is in line with the findings by Damluji et al. [52] that found similar results in femoral operators overall. This suggests that there is a systemic problem with femoral training that needs to be addressed so that safe vascular access at any site can be taught.

The phenomenon of Campeau Radial Paradox was central to our survey. This term was coined by Azzalini et al. in 2015 after conducting a retrospective analysis of two historical cohorts of patients undergoing PCI at the Montreal Heart Institute during the periods of 19961998 and 20062008 [1, 42]. They concluded that while TRA has reduced vascular complication rates at an individual level, it has led to increased rates at a population level driven primarily by TFA-related complications. This was later challenged by Hulme et al. in a large retrospective analysis of the British Cardiovascular Intervention Society (BCIS), showing that there were no significant differences in 30-day mortality or complication rates between centers, regardless of femoral proportion per center [1, 19]. Respondents were asked regarding their belief in the proposed Campeau paradox; 62% of trainees and as many as 67% of trainers believe that the increased and abrupt adoption of TRA has resulted in a paradoxical spike in complications at the population level due to declining TFA expertise.

Our study sheds light on the interplay between increasingly stronger recommendations for TRA and the possible resultant decline in the quality of TFA training. While the European Society of Cardiology guidelines [2015] recommend radial over femoral approach [53], the American Heart Association guidelines [2015] did not recommend one access site over the other [54]. However, in 2018 a radial-first approach was strongly recommended by the AHA [55]. Faced with this increasing emphasis on TRA as the preferred choice, the apprehensions of fellows and faculty regarding lower exposure to TFA remained largely undocumented prior to this analysis. Of the trainers responding to our survey, 37.5% were very concerned and 31% were somewhat concerned about the declining exposure of trainees to TFA and related complications. Moreover, 65% of the trainers believed their trainees will not achieve the same level of expertise in TFA as their predecessors.

TFA remains a much-needed tool in the arsenal of invasive and interventional cardiologists. A 2018 Cochrane database review of 28 RCTs found there was a significantly higher incidence of crossover with transradial approach compared to TFA [56]. Thus TRA may be a preferred route of access but a sufficiently high skill level in TFA needs to be maintained in current and future training fellows. TFA remains relevant due to the ever-evolving need for large bore access. When asked about the future of TFA, most of our respondents [77% trainees and 81% trainers] believed that while TFA frequency will decline, it will continue to remain relevant as a major access point.

A major argument in favor of TRA has come from trials including RIVAL, MATRIX and RIFLE-STEACS revealing lower risk of bleeding and mortality in TRA compared to TFA [10, 11, 15]. However, the more recent SAFARI-STEMI trial did not show significant difference in 30-day mortality or bleeding complications in TRA or TFA in primary PCI [5]. This suggests that adequately trained operators can attain similar results with TRA or TFA for PCI.

We believe that access preference should take root in an understanding of the purpose of each approach and when each should be favored [57]. To optimize practice in acquiring femoral access, educational programs for trainees should ensure incorporation of formal teaching, workshops, and simulators geared toward the femoral approach [58]. Adequate training should also be provided in the use of fluoroscopy, ultrasound guidance and vascular closure devices, most of which have been reported to increase safety, comfort and convenience with TFA [59,63]. As recommended by the American Heart Association, femoral access skills can be maintained through peripheral vascular, structural cardiac, or ventricular assist device insertion procedure [27]. We believe that the apprehensions regarding the quality of TFA training expressed by trainers across the country mandate a structured approach towards ensuring adequate education in femoral access for all trainees.

Our study had a few limitations. Survey-based designs are vulnerable to biases, but since our aim was to gauge subjective parameters, we believe it was the appropriate investigative modality in absence of a better alternative. Another limitation is the small sample size. Despite having a smaller sample, our results suggesting increased use and familiarity of transradial access are similar to a larger recent survey studying radial access practices (449 US interventional cardiologists) [64]. This study recognized the heterogeneity in practices for transradial access. Whether our results can be extrapolated to the majority of US PCI centers cannot be fully determined at this time.

## Conclusion

The shift from TFA to radial first has resulted in significant concern among cardiovascular fellows-in training and the faculty regarding training in TFA. Cardiovascular training programs must be cognizant of this issue and should devise methods to assure optimal training of fellows in gaining TFA and managing femoral access-related complications. Routine use of ultrasound for TFA must be encouraged. A larger study with objective parameters is required to assess if outcomes in patients undergoing TFA currently and in the near future are similar or have changed compared to when TFA was being used more commonly.

## Data Availability

The datasets used and/or analyzed during the current study available from the corresponding author on reasonable request.

## Abbreviations

TRA: Trans Radial Access
TFA: Trans Femoral Access
CV: Cardiovascular
PCI: Percutaneous Coronary Intervention
SPSS: Statistical Package for Social Sciences
US: United States
RCT: Randomized Controlled Trial
ESC: European Society of Cardiology
AHA: American Heart Association
ACC: American College of Cardiology
